# Supplementary material for: Biometric characteristics of winter rape plants (Brassica napus L.) before harvest in the soil and climatic conditions of north-eastern Poland
Source: PLoS One. 2023 Aug 16;18(8):e0289947. doi: 10.1371/journal.pone.0289947 (PMC10431616; doi:10.1371/journal.pone.0289947)
Supplement: S1 Table — (DOCX) [file pone.0289947.s001.docx]

**S1 Table. Preparations used in the experiment**

| **METHODS OF USING PREPARATIONS** | **Time of application of preparations in the BBCH phase** | **DOSE** |
| --- | --- | --- |
| **variant 1 – control object** | without using the preparations | - |
| **variant 2- organic preparation (Ugmax)** | I - in autumn before sowing seeds  II - in spring after the start of vegetation: the beginning of the development of side shoots (BBCH 21-36) | 0.9 dm^3^·ha^-1^ |
| **variant 3 - biostimulant containing 13.0% of P₂0₅ and 5.0% of K₂O** | I - in autumn, 4-6 leaves phase (BBCH 13-15)  II – in spring after the start of vegetation (BBCH 28-30) | 1.0 dm^3^·ha^-1^ |
| **variant 4 - biostimulant containing silicon** | I - in autumn, 4-6 leaves phase (BBCH 13-15)  II – dense green flower bud in spring (BBCH 51) | 0.5 dm^3^·ha^-1^ |
