# Supplementary material for: Biometric characteristics of winter rape plants (Brassica napus L.) before harvest in the soil and climatic conditions of north-eastern Poland
Source: PLoS One. 2023 Aug 16;18(8):e0289947. doi: 10.1371/journal.pone.0289947 (PMC10431616; doi:10.1371/journal.pone.0289947)
Supplement: S2 Table — (DOCX) [file pone.0289947.s002.docx]

**S2 Table. Mineral fertilization used in the experiment**

| **Date of fertilization** | **Type of fertilization** | **Doses** |
| --- | --- | --- |
| **before sowing** | phosphorus-potassium fertilization | 40 kg P·ha^-1^  110 kg K·ha^-1^  first dose 40 kg N·ha^-1^ |
| **during**  **autumn growth and development** | Lubofos | dose 600 kg containing:  21 kg N·ha^-1^  26.4 kg P·ha^-1^  92.1 kg K·ha^-1^  34.8 kg S·ha^-1^  1.2 kg B·ha^-1^ |
|  | ammonium sulphate  triple superphosphate  potassium salt | 55.9 kg·ha^-1^ (19 kg N·ha^-1^),  29.6 kg·ha^-1^ (13.6 kg P·ha^-1^)  29 kg·ha^-1^ (17.9 kg K·ha^-1^) |
| **in spring before vegetation starts (BBCH 28-30)** | ammonium nitrate  ammonium sulphate | 255.5 kg·ha^-1^ (86.9 kg N·ha^-1^)  62.5 kg·ha^-1^ (13.1 kg N·ha^-1^ +15 kg S·ha^-1^) |
| **at the beginning of budding (BBCH 50)** | ammonium nitrate | 176.5 kg·ha^-1^ (60 kg N·ha^-1^) |
