# Supplementary material for: Biometric characteristics of winter rape plants (Brassica napus L.) before harvest in the soil and climatic conditions of north-eastern Poland
Source: PLoS One. 2023 Aug 16;18(8):e0289947. doi: 10.1371/journal.pone.0289947 (PMC10431616; doi:10.1371/journal.pone.0289947)
Supplement: S3 Table — (DOCX) [file pone.0289947.s003.docx]

**S3 Table. Type of chemical protection treatments**

| **Active substance in the agent used** | **Dose of the agent used** | **Development phases** | **acc. to the BBCH scale** |
| --- | --- | --- | --- |
| **HERBICIDES** | | | |
| **clomazone** | 0.25 dm^3^·ha^-1^ | immediately after sowing on the soil | 00 BBCH |
| **fluazifop-P-butyl** | 2.0 dm^3^·ha^-1^ | phase of 3-4 leaves | 13-14 BBCH |
| **INSECTICIDES** | | | |
| **thiacloprid deltamethrin** | 0.6 dm^3^·ha^-1^ | 1 treatment – growth of the main shoot,  2 treatment – development of flower buds  3 treatment - flowering | 30 BBCH  50-58 BBCH  60-69 BBCH |
| **FUNGICIDES** | | | |
| **tebuconazole** | 0.75 dm^3^·ha^-1^ | phase of 4-8 leaves of rape | 14-18 BBCH |
| **fluopyram**  **prothioconazole** | 1.0 dm^3^·ha^-1^ | beginning of flowering | 61 BBCH |
| **prochloraz** | 1.0 dm^3^·ha^-1^ | fall phase of the first flower petals | 1. BBCH |
