# Supplementary material for: Biometric characteristics of winter rape plants (Brassica napus L.) before harvest in the soil and climatic conditions of north-eastern Poland
Source: PLoS One. 2023 Aug 16;18(8):e0289947. doi: 10.1371/journal.pone.0289947 (PMC10431616; doi:10.1371/journal.pone.0289947)
Supplement: S4 Table — (DOCX) [file pone.0289947.s004.docx]

**S4 Table.** **The value of the Sielianinow hydrothermal coefficient**

|  | **Value of the Sielianinow hydrothermal coefficient *** | | | | | | | | |
| --- | --- | --- | --- | --- | --- | --- | --- | --- | --- |
|  | **VIII** | **IX** | **X** | **III** | **IV** | **V** | **VI** | **VII** | **Mean** |
| **I year of research (2018-2019)**  **II year of research**  **(2019-2020)**  **III year of research**  **(2020-2021)** | 1.19  2.20  0.90 | 1.72  1.22  2.50 | 2.42  0.89  4.39 | 3.13  1.31  3.55 | 0.60  0.70  6.36 | 4.49  5.42  2.38 | 1.68  6.14  1.66 | 1.60  3.56  2.20 | 2.10  2.68  2.99 |

* Coefficient value [14]: Extremely dry (ss) k≤0.4; Very dry (bs) 0.4-0.7; Dry (s) 0.7-1.0; Rather dry (ds) 1.0<k≤1.3; Optimal (o) 1.3<k≤1.6; Rather wet (dw) 1.6<k≤2.0; Wet (w) 2.0<k≤2.5; Very wet (bw) 2.5<k≤3.0; Extremely wet (sw) k>3.0
