# Supplementary material for: Biometric characteristics of winter rape plants (Brassica napus L.) before harvest in the soil and climatic conditions of north-eastern Poland
Source: PLoS One. 2023 Aug 16;18(8):e0289947. doi: 10.1371/journal.pone.0289947 (PMC10431616; doi:10.1371/journal.pone.0289947)
Supplement: S5 Table — (DOCX) [file pone.0289947.s005.docx]

**S5 Table.** **Climatic data 2019-2021 (Agricultural Experimental Station, Poland)**

|  | Precipitation (mm) | | | | **Air temperature (^o^C)** | | | |
| --- | --- | --- | --- | --- | --- | --- | --- | --- |
|  | I year | II year | III year | Multiyear total  (1996-2010) | I year | II year | III year | **Multiyear mean**  **(1996-2010)** |
| **VIII** | 24.5 | 43.9 | 18.2 | 59.9 | 20.6 | 19.9 | 20.2 | 18.5 |
| **IX** | 27.4 | 17.4 | 38.8 | 42.3 | 15.9 | 14.2 | 15.5 | 13.5 |
| **X** | 23.3 | 9.5 | 52.7 | 24.2 | 9.6 | 10.7 | 12.0 | 7.9 |
| **XI** | 9.8 | 17.8 | 34.0 | 20.2 | 3.3 | 6.1 | 5.0 | 4.0 |
| **XII** | 9.0 | 29.1 | 34.0 | 18.6 | 0.4 | 2.9 | -1.0 | -0.1 |
| **I** | 7.9 | 12.9 | 22.6 | 19.0 | -3.0 | 1.9 | -1.9 | -3.2 |
| **II** | 4.7 | 26.8 | 10.4 | 16.0 | 2.2 | 2.9 | -2.5 | -2.3 |
| **III** | 15.0 | 5.9 | 9.6 | 18.3 | 4.8 | 4.5 | 2.7 | 2.4 |
| **IV** | 5.9 | 6.0 | 42.0 | 33.6 | 9.8 | 8.6 | 6.6 | 8.0 |
| **V** | 59.8 | 63.5 | 29.5 | 58.3 | 13.3 | 11.7 | 12.4 | 13.5 |
| **VI** | 35.9 | 118.5 | 33.8 | 59.6 | 21.4 | 19.3 | 20.4 | 17.0 |
| **VII** | 29.7 | 67.7 | 50.0 | 57.5 | 18.5 | 19.0 | 22.7 | 19.7 |
| **Mean** | **244.0** | 419.0 | 375.6 | 427.5 | 9.7 | 10.1 | 9.3 | 8.2 |
