# Supplementary material for: Biometric characteristics of winter rape plants (Brassica napus L.) before harvest in the soil and climatic conditions of north-eastern Poland
Source: PLoS One. 2023 Aug 16;18(8):e0289947. doi: 10.1371/journal.pone.0289947 (PMC10431616; doi:10.1371/journal.pone.0289947)
Supplement: S6 Table — (DOCX) [file pone.0289947.s006.docx]

**S6 Table.** **Biometric characteristics of plants depending on the factors of experience**

| **Methods of using preparations** | | **Cultivars** | | | **Mean** |
| --- | --- | --- | --- | --- | --- |
|  |  | population | restored hybrid with a traditional type of growth | restored hybrid with a semi-dwarf type of growth |  |
|  | | | | | |
| **Plant height (cm)** | | | | | |
| 1. | Variant control | 133.7 | 120.7 | 119.4 | **124.6** |
| 2. | Organic preparation containing microorganisms as well as micro and macro elements | 142.1 | 127.7 | 123.4 | **131.1** |
| 3. | Biostimulant containing 13.0% of P₂0₅ and 5.0% of potassium oxide (K₂O) | 137.0 | 123.4 | 121.0 | **127.4** |
| 4. | Biostimulant containing silicon | 139.2 | 131.1 | 122.0 | **128.6** |
| **Mean** | | **138.0** | **124.3** | **121.5** | - |
| **LSD_0.05_ for:**  *cultivars*  *methods of using preparations*  *interaction: cultivars x methods of using preparations* | | | | | 0.6  0.8  1.4 |
| **Height of the first productive branching (cm)** | | | | | |
| 1. | Variant control | 45.4 | 38.0 | 36.8 | **40.1** |
| 2. | Organic preparation containing microorganisms as well as micro and macro elements | 49.1 | 40.9 | 40.2 | **43.4** |
| 3. | Biostimulant containing 13.0% of P₂0₅ and 5.0% of potassium oxide (K₂O) | 47.9 | 40.1 | 38.9 | **42.3** |
| 4. | Biostimulant containing silicon | 49.0 | 40.3 | 39.1 | **42.8** |
| **Mean** | | **47.8** | **39.8** | **38.8** | - |
| **LSD_0.05_ for:**  *cultivars*  *methods of using preparations*  *interaction: cultivars x methods of using preparations* | | | | | 0.5  0.7  1.2 |
| **Number of productive branches (pcs.)** | | | | | |
| 1. | Variant control | 3.9 | 4.2 | 4.0 | **4.0** |
| 2. | Organic preparation containing microorganisms as well as micro and macro elements | 5.1 | 5.1 | 5.0 | **5.1** |
| 3. | Biostimulant containing 13.0% of P₂0₅ and 5.0% of potassium oxide (K₂O) | 4.1 | 4.5 | 4.1 | **4.2** |
| 4. | Biostimulant containing silicon | 4.5 | 4.7 | 4.7 | **4.6** |
| **Mean** | | **4.4** | **4.6** | **4.4** | **-** |
| **LSD_0.05_ for:**  *cultivars*  *methods of using preparations*  *interaction: cultivars x methods of using preparations* | | | | | 0.1  0.1  0.2 |
| **Number of siliques per plant (pcs.)** | | | | | |
| 1. | Variant control | 130.6 | 136.8 | 128.9 | **132.1** |
| 2. | Organic preparation containing microorganisms as well as micro and macro elements | 143.6 | 150.2 | 144.2 | **146.0** |
| 3. | Biostimulant containing 13.0% of P₂0₅ and 5.0% of potassium oxide (K₂O) | 131.2 | 133.1 | 132.0 | **132.1** |
| 4. | Biostimulant containing silicon | 139.3 | 145.8 | 139.6 | **141.6** |
| **Mean** | | **136.2** | **141.5** | **136.2** | **-** |
| **LSD_0.05_ for:**  *cultivars*  *methods of using preparations*  *interaction: cultivars x methods of using preparations* | | | | | 1.7  1.5  2.6 |
| **Length of the pods (cm)** | | | | | |
| 1. | Variant control | 7.0 | 7.2 | 6.9 | **7.0** |
| 2. | Organic preparation containing microorganisms as well as micro and macro elements | 7.8 | 8.1 | 7.8 | **7.9** |
| 3. | Biostimulant containing 13.0% of P₂0₅ and 5.0% of potassium oxide (K₂O) | 7.0 | 7.5 | 7.0 | **7.2** |
| 4. | Biostimulant containing silicon | 7.5 | 7.7 | 7.5 | **7.6** |
| **Mean** | | **7.3** | **7.6** | **7.3** | **-** |
| **LSD_0.05_ for:**  *Cultivars*  *methods of using preparations*  *interaction: odmiany x methods of using preparations* | | | | | 0.1  0.1  0.1 |
| **Thickness of the stem at the base (mm)** | | | | | |
| 1. | Variant control | 13.80 | 13.62 | 13.91 | **13.78** |
| 2. | Organic preparation containing microorganisms as well as micro and macro elements | 15.04 | 14.39 | 15.21 | **14.88** |
| 3. | Biostimulant containing 13.0% of P₂0₅ and 5.0% of potassium oxide (K₂O) | 14.82 | 13.98 | 14.11 | **14.30** |
| 4. | Biostimulant containing silicon | 14.68 | 13.97 | 14.24 | **14.30** |
| **Mean** | | **14.59** | **13.99** | **14.37** | **-** |
| **LSD_0.05_ for:**  *cultivars*  *methods of using preparations*  *interaction: cultivars x methods of using preparations* | | | | | 0.17  0.24  0.42 |
