# Supplementary material for: Biometric characteristics of winter rape plants (Brassica napus L.) before harvest in the soil and climatic conditions of north-eastern Poland
Source: PLoS One. 2023 Aug 16;18(8):e0289947. doi: 10.1371/journal.pone.0289947 (PMC10431616; doi:10.1371/journal.pone.0289947)
Supplement: S7 Table — (DOCX) [file pone.0289947.s007.docx]

**S7 Table.** **Biometric characteristics of plants depending on years of research and morphotypes**

| **Years** | **Cultivars** | | | **Mean** |
| --- | --- | --- | --- | --- |
|  | population | restored hybrid with a traditional type of growth | restored hybrid with a semi-dwarf type of growth |  |
| **Plant height (cm)** | | | | |
| I | 131.5 | 121.5 | 120.0 | **124.3** |
| II | 142.8 | 126.8 | 122.0 | **130.5** |
| III | 139.8 | 124.6 | 122.3 | **128.9** |
| **Mean** | **138.0** | **124.3** | **121.5** | **-** |
| **LSD_0.05_ for:**  *cultivars*  *years*  *interaction: cultivars x years* | | | | 0.6  0.6  1.1 |
| **Height of the first productive branching (cm)** | | | | |
| I | 40.7 | 35.7 | 34.1 | **36.8** |
| II | 57.2 | 43.4 | 42.5 | **47.7** |
| III | 45.6 | 40.3 | 39.7 | **41.9** |
| **Mean** | **47.8** | **39.8** | **38.8** | - |
| **LSD_0.05_ for:**  *cultivars*  *years*  *interaction: cultivars x years* | | | | 0.5  0.5  0.8 |
| **Number of productive branches (pcs.)** | | | | |
| I | 3.9 | 3.9 | 3.7 | **3.8** |
| II | 5.0 | 5.4 | 5.2 | **5.2** |
| III | 4.4 | 4.6 | 4.5 | **4.5** |
| **Mean** | **4.4** | **4.6** | **4.4** | **-** |
| **LSD_0.05_ for:**  *cultivars*  *years*  *interaction: cultivars x years* | | | | 0.1  0.1  0.2 |
| **Number of siliques per plant (pcs.)** | | | | |
| I | 125.9 | 130.9 | 124.8 | **127.2** |
| II | 150.4 | 148.8 | 146.9 | **148.7** |
| III | 132.2 | 144.6 | 136.9 | **137.9** |
| **Mean** | **136.2** | **141.5** | **136.2** | **-** |
| **LSD_0.05_ for:**  *cultivars*  *years*  *interaction: cultivars x years* | | | | 1.7  1.7  2.9 |
| **Length of the pods (cm)** | | | | |
| I | 6.7 | 6.9 | 6.6 | **6.7** |
| II | 8.2 | 8.5 | 7.9 | **8.2** |
| III | 7.1 | 7.6 | 7.4 | **7.3** |
| **Mean** | **7.3** | **7.6** | **7.3** | **-** |
| **LSD_0.05_ for:**  *cultivars*  *years*  *interaction: cultivars x years* | | | | 0.1  0.1  0.2 |
| **Thickness of the stem at the base (mm)** | | | | |
| I | 13.63 | 13.04 | 13.66 | **13.44** |
| II | 15.74 | 15.18 | 15.48 | **15.47** |
| III | 14.39 | 13.75 | 13.97 | **14.04** |
| **Mean** | **14.59** | **13.99** | **14.37** | **-** |
| **LSD_0.05_ for:**  *cultivars*  *years*  *interaction: cultivars x years* | | | | 0.17  0.17  0.29 |
