# Supplementary material for: Biometric characteristics of winter rape plants (Brassica napus L.) before harvest in the soil and climatic conditions of north-eastern Poland
Source: PLoS One. 2023 Aug 16;18(8):e0289947. doi: 10.1371/journal.pone.0289947 (PMC10431616; doi:10.1371/journal.pone.0289947)
Supplement: S8 Table — (DOCX) [file pone.0289947.s008.docx]

**S8 Table. Biometric characteristics of plants depending on the years of research and the types of preparations used**

| **Methods of using preparations** | ***Years*** | | | **Mean** |
| --- | --- | --- | --- | --- |
|  | **2018-2019** | **2019-2020** | **2020-2021** |  |
| **Plant height (cm)** | | | | |
| 1. Variant control | 120.4 | 127.3 | 126.2 | **124.6** |
| 2. Organic preparation containing microorganisms as well as micro and macro elements | 128.0 | 133.6 | 131.6 | **131.1** |
| 3. Biostimulant containing 13.0% of P₂0₅ and 5.0% of potassium oxide (K₂O) | 123.6 | 129.7 | 128.9 | **127.4** |
| 4. Biostimulant containing silicon | 125.2 | 131.5 | 129.0 | **128.6** |
| **Mean** | **124.3** | **130.5** | **128.9** | **-** |
| **LSD_0.05_ for:**  *years*  *methods of using preparations*  interaction: *years* x *methods of using preparations* | | | | 0.6  0.8  1.4 |
| **Height of the first productive branching (cm)** | | | | |
| 1. Variant control | 34.5 | 46.2 | 39.5 | **40.1** |
| 2. Organic preparation containing microorganisms as well as micro and macro elements | 38.3 | 48.6 | 43.2 | **43.4** |
| 3. Biostimulant containing 13.0% of P₂0₅ and 5.0% of potassium oxide (K₂O) | 37.1 | 47.6 | 42.2 | **42.3** |
| 4. Biostimulant containing silicon | 37.4 | 48.4 | 42.6 | **42.8** |
| **Mean** | **36.8** | **47.7** | **41.9** | - |
| **LSD_0.05_ for:**  *years*  *methods of using preparations*  interaction: *years* x *methods of using preparations* | | | | 0.5  0.7  r.n. |
| **Number of productive branches (pcs.)** | | | | |
| 1. Variant control | 3.3 | 4.7 | 4.1 | **4.0** |
| 2. Organic preparation containing microorganisms as well as micro and macro elements | 4.4 | 5.9 | 5.0 | **5.1** |
| 3. Biostimulant containing 13.0% of P₂0₅ and 5.0% of potassium oxide (K₂O) | 3.4 | 4.7 | 4.6 | **4.3** |
| 4. Biostimulant containing silicon | 4.2 | 5.4 | 4.3 | **4.6** |
| **Mean** | **3.8** | **5.2** | **4.5** | **-** |
| **LSD_0.05_ for:**  *years*  methods of using preparations  interaction: *years* x *methods of using preparations* | | | | 0.1  0.1  0.2 |
| **Number of siliques per plant (pcs.)** | | | | |
| 1. Variant control | 122.2 | 144.0 | 130.1 | **132.1** |
| 2. Organic preparation containing microorganisms as well as micro and macro elements | 134.1 | 154.9 | 148.9 | **146.0** |
| 3. Biostimulant containing 13.0% of P₂0₅ and 5.0% of potassium oxide (K₂O) | 121.9 | 143.6 | 130.8 | **132.1** |
| 4. Biostimulant containing silicon | 130.6 | 152.3 | 141.7 | **141.6** |
| **Mean** | **127.2** | **148.7** | **137.9** | **-** |
| **LSD_0.05_ for:**  *years*  *methods of using preparations*  interaction: *years* x *methods of using preparations* | | | | 1.7  1.5  2.6 |
| **Length of the pods (cm)** | | | | |
| 1. Variant control | 6.2 | 8.0 | 7.0 | **7.0** |
| 2. Organic preparation containing microorganisms as well as micro and macro elements | 7.3 | 8.5 | 7.8 | **7.9** |
| 3. Biostimulant containing 13.0% of P₂0₅ and 5.0% of potassium oxide (K₂O) | 6.3 | 7.9 | 7.2 | **7.2** |
| 4. Biostimulant containing silicon | 7.1 | 8.4 | 7.4 | **7.6** |
| **Mean** | **6.7** | **8.2** | **7.3** | **-** |
| **LSD_0.05_ for:**  *years*  *methods of using preparations*  interaction: *years* x *methods of using preparations* | | | | 0.1  0.1  0.1 |
| **Thickness of the stem at the base (mm)** | | | | |
| 1. Variant control | 12.86 | 14.90 | 13.58 | **13.78** |
| 2. Organic preparation containing microorganisms as well as micro and macro elements | 14.19 | 15.98 | 14.48 | **14.88** |
| 3. Biostimulant containing 13.0% of P₂0₅ and 5.0% of potassium oxide (K₂O); | 13.38 | 15.42 | 14.11 | **14.30** |
| 4. Biostimulant containing silicon | 13.34 | 15.57 | 13.98 | **14.30** |
| **Mean** | **13.44** | **15.47** | **14.04** | **-** |
| **LSD_0.05_ for:**  *years*  *methods of using preparations*  interaction: *years* x *methods of using preparations* | | | | 0.17  0.24  0.42 |
