# Supplementary material for: Biometric characteristics of winter rape plants (Brassica napus L.) before harvest in the soil and climatic conditions of north-eastern Poland
Source: PLoS One. 2023 Aug 16;18(8):e0289947. doi: 10.1371/journal.pone.0289947 (PMC10431616; doi:10.1371/journal.pone.0289947)
Supplement: Attachment — Submitted filename: PONE-D-23-15441_reviewer.pdf [file pone.0289947.s009.pdf]

PLOS ONE

Biometric characteristics of winter rape plants (Brassica napus l.) before harvest in the soil and climatic conditions of north-eastern Poland, depending on the bio-stimulative preparations used

--Manuscript Draft--

**Manuscript Number:**	PONE-D-23-15441
**Article Type:**	Research Article
**Full Title:**	Biometric characteristics of winter rape plants (Brassica napus l.) before harvest in the soil and climatic conditions of north-eastern Poland, depending on the bio-stimulative preparations used
**Short Title:**	Biometric characteristics of winter rape plants (Brassica napus l.) before harvest in the soil and climatic
**Corresponding Author:**	ANNA SIKORSKA<br>PUZ im Ignacego Mościckiego w Ciechanowie: Panstwowa Uczelnia Zawodowa im Ignacego Moscickiego w Ciechanowie<br>Ciechanów, CIECHANÓW POLAND
**Keywords:**	Ugmax, biostimulant, biofertiliser, winter rape, biometric characteristics
**Abstract:**	The research was based on a field experiment carried out in the Agricultural Experimental Station in the climatic and soil conditions of north-eastern Poland. The factors of the experiment were: I – morphotypes of winter oilseed rape: population, restored hybrid with a traditional type of growth, restored hybrid with a semi-dwarf type of growth. II – methods of using preparations: variant (1) - control – without using preparations; variant (2) – an organic preparation containing microorganisms as well as micro and macro elements (Ugmax); variant (3) – a biostimulant containing 13.0% of P_20_5 and 5.0% of potassium oxide (K_2O); variant (4) – a biostimulant containing silicon. The research showed that the population morphotype was characterized by the highest plant height before harvesting, the height to the first branching, the thickness of the stem at the base, and the long-stemmed hybrid had the largest number of productive branches and siliques on the plant. The organic preparation containing microorganisms as well as micro and macro-elements, applied in the autumn before sowing seeds and in the spring after the start of vegetation, had the most beneficial effect on the biometric characteristics of plants before harvesting
**Order of Authors:**	ANNA SIKORSKA, prof.
	MAREK GUGAŁA, prof.
	KRYSTYNA ZARZECKA, prof.
	IWONA MYSTKOWSKA, prof.
	Agnieszka Ginter, dr
	PAVOL FINDURA
	Miroslav Pristavka, dr
**Additional Information:**	
**Question**	**Response**
**Financial Disclosure**<br><br>Enter a financial disclosure statement that describes the sources of funding for the work included in this submission. Review the submission guidelines for detailed requirements. View published research articles from *PLOS ONE* for specific	The results of the research carried out under the research theme No. 32/20/B were financed from the science grant granted by the Ministry of Science and Higher Education.

Powered by Editorial Manager® and ProduXion Manager® from Aries Systems Corporation

examples.<br><br>This statement is required for submission and **will appear in the published article** if the submission is accepted. Please make sure it is accurate.<br><br>**Unfunded studies**<br>Enter: *The author(s) received no specific funding for this work.*<br><br>**Funded studies**<br>Enter a statement with the following details:<br>• Initials of the authors who received each award<br>• Grant numbers awarded to each author<br>• The full name of each funder<br>• URL of each funder website<br>• Did the sponsors or funders play any role in the study design, data collection and analysis, decision to publish, or preparation of the manuscript?<br>• **NO** - Include this sentence at the end of your statement: *The funders had no role in study design, data collection and analysis, decision to publish, or preparation of the manuscript.*<br>• **YES** - Specify the role(s) played.<br><br>* typeset	
**Competing Interests**<br><br>Use the instructions below to enter a competing interest statement for this submission. On behalf of all authors, disclose any competing interests that could be perceived to bias this work—acknowledging all financial support and any other relevant financial or non-financial competing interests.<br><br>This statement is required for submission and **will appear in the published article** if the submission is accepted. Please make sure it is accurate and that any funding sources listed in your Funding Information later in the submission form are also declared in your Financial Disclosure statement.	he authors have declared that no competing interests exist.

View published research articles from *PLOS ONE* for specific examples.<br><br>**NO authors have competing interests**<br><br>Enter: *The authors have declared that no competing interests exist.*<br><br>**Authors with competing interests**<br><br>Enter competing interest details beginning with this statement:<br><br>*I have read the journal's policy and the authors of this manuscript have the following competing interests: [insert competing interests here]*<br><br>* typeset	
**Ethics Statement**<br><br>Enter an ethics statement for this submission. This statement is required if the study involved:<br><br>• Human participants<br>• Human specimens or tissue<br>• Vertebrate animals or cephalopods<br>• Vertebrate embryos or tissues<br>• Field research<br><br>Write "N/A" if the submission does not require an ethics statement.<br><br>General guidance is provided below. Consult the submission guidelines for detailed instructions. **Make sure that all information entered here is included in the Methods section of the manuscript.**	NOT Animal Research

Powered by Editorial Manager® and ProduXion Manager® from Aries Systems Corporation

**Format for specific study types**<br><br>**Human Subject Research (involving human participants and/or tissue)**<br>• Give the name of the institutional review board or ethics committee that approved the study<br>• Include the approval number and/or a statement indicating approval of this research<br>• Indicate the form of consent obtained (written/oral) or the reason that consent was not obtained (e.g. the data were analyzed anonymously)<br><br>**Animal Research (involving vertebrate animals, embryos or tissues)**<br>• Provide the name of the Institutional Animal Care and Use Committee (IACUC) or other relevant ethics board that reviewed the study protocol, and indicate whether they approved this research or granted a formal waiver of ethical approval<br>• Include an approval number if one was obtained<br>• If the study involved *non-human primates*, add *additional details* about animal welfare and steps taken to ameliorate suffering<br>• If anesthesia, euthanasia, or any kind of animal sacrifice is part of the study, include briefly which substances and/or methods were applied<br><br>**Field Research**<br><br>Include the following details if this study involves the collection of plant, animal, or other materials from a natural setting:<br>• Field permit number<br>• Name of the institution or relevant body that granted permission	
**Data Availability**<br><br>Authors are required to make all data underlying the findings described fully available, without restriction, and from the time of publication. PLOS allows rare exceptions to address legal and ethical concerns. See the PLOS Data Policy and FAQ for detailed information.	Yes - all data are fully available without restriction

A Data Availability Statement describing where the data can be found is required at submission. Your answers to this question constitute the Data Availability Statement and **will be published in the article**, if accepted.<br><br>**Important:** Stating 'data available on request from the author' is not sufficient. If your data are only available upon request, select 'No' for the first question and explain your exceptional situation in the text box.<br><br>Do the authors confirm that all data underlying the findings described in their manuscript are fully available without restriction?	
**Describe where the data may be found in full sentences. If you are copying our sample text, replace any instances of XXX with the appropriate details.**<br><br>• If the data are **held or will be held in a public repository**, include URLs, accession numbers or DOIs. If this information will only be available after acceptance, indicate this by ticking the box below. For example: *All XXX files are available from the XXX database (accession number(s) XXX, XXX.).*<br>• If the data are all contained **within the manuscript and/or Supporting Information files**, enter the following: *All relevant data are within the manuscript and its Supporting Information files.*<br>• If neither of these applies but you are able to provide **details of access elsewhere**, with or without limitations, please do so. For example:<br><br>*Data cannot be shared publicly because of [XXX]. Data are available from the XXX Institutional Data Access / Ethics Committee (contact via XXX) for researchers who meet the criteria for access to confidential data.*<br><br>*The data underlying the results presented in the study are available from (include the name of the third party*	-

Powered by Editorial Manager® and ProduXion Manager® from Aries Systems Corporation

and contact information or URL).<br>• This text is appropriate if the data are owned by a third party and authors do not have permission to share the data.<br><br>* typeset	
Additional data availability information:	Tick here if your circumstances are not covered by the questions above and you need the journal's help to make your data available.

Powered by Editorial Manager® and ProduXion Manager® from Aries Systems Corporation

Click here to access/download;Manuscript;Manuscript.docx

Biometric characteristics of winter rape plants (*Brassica napus* l.) before harvest in the soil and climatic conditions of north-eastern Poland, depending on the bio-stimulative preparations used

Anna Sikorska[1], Marek Gugała[2], Krystyna Zarzecka[2], Iwona Mystkowska[3], Agnieszka Ginter[2], Pavol Findura[4], Miroslav Pristavka[4]

[1]*Department of Agriculture, Vocational State School of Ignacy Mościcki in Ciechanów, ul. Narutowicza 9, 06-400 Ciechanów, Poland; anna.sikorska@puzim.edu.pl*
[2]*Faculty of Agrobioengineering and Animal Husbandry, University of Natural Sciences and Humanities in Siedlce, ul. Prusa 14, 08-110 Siedlce, Poland;, gugala@uph.edu.pl; kzarzecka@uph.edu.pl, agnieszka.ginter@uph.edu.pl*
[3] *Department of Dieteties, Pope John Paul II State School of Higher Education in Biała Podlaska, ul. Sidorska 95/97, 21-500 Biala Podlaska, Poland, imystkowska@op.pl*
[4] *Department of Machines and Production Biosystems, Faculty of Engineering, Slovak University of Agriculture in Nitra, Tr. A. Hlinku 2, 949 76 Nitra, Slovakia*

Abstract:

The research was based on a field experiment carried out in the Agricultural Experimental Station in the climatic and soil conditions of north-eastern Poland. The factors of the experiment were: I – morphotypes of winter oilseed rape: population, restored hybrid with a traditional type of growth, restored hybrid with a semi-dwarf type of growth. II – methods of using preparations: variant (1) - control – without using preparations; variant (2) – an organic preparation containing microorganisms as well as micro and macro elements (Ugmax); variant (3) – a biostimulant containing 13.0% of $P_2\ 0_5$ and 5.0% of potassium oxide (K_2 O); variant (4) – a biostimulant containing silicon. The research showed that the population morphotype was characterized by the highest plant height before harvesting, the height to the first branching, the thickness of the stem at the base, and the long-stemmed hybrid had the largest number of productive branches and siliques on the plant. The organic preparation containing microorganisms as well as micro and macro-elements, applied in the autumn before sowing seeds and in the spring after the start of vegetation, had the most beneficial effect on the biometric characteristics of plants before harvesting.

Keywords: Ugmax, biostimulant, biofertiliser, winter rape, biometric characteristics

INTRODUCTION

The use of simplifications in tillage and crop rotation results in the impoverishment of the soil environment in beneficial microorganisms. Many authors (Al-Taweil et al. 2009, Pietkiewicz et al. 2004) believe that continuous and excessive use of chemical fertilizers and plant protection products causes ecological and health hazards, and also worsens the physical and chemical condition of the soil, contributing as a result to reduction in crop yield and quality.

Biostimulants and bioprotectants are natural preparations that have gained a lot of interest in recent years due to their role in improving plant growth and yield and reducing the impact of abiotic and biotic stresses (Mrid et al. 2021). Du Jardin, 2015 gives a division of biostimulants into individual categories: humic acid and fulvic acid, proteolytic substances, seaweed extracts, chitosan, inorganic compounds, as well as beneficial fungi and bacteria.

Organic sources play an important role in improving soil fertility and crop productivi[illegible] Biopreparations stimulate the growth and development of crops, protect against pathogens and enrich the soil with nutrients, while biofertilizers contain selected microorganisms that, through intersection in the rhizosphere, improve soil quality and increase the absorption of hard-to-reach nutrients by plants. Therefore, more and more often in agricultural practice, e.g. soil fertilizers, which increase the content of humus in the soil, accelerate the decomposition of mineral and organic fertilizers and increase the availability of nutrients for plants (Gobin 2012, Findura et al. 2022).

One of the biofertilizers used in the experiment was the Ugmax preparation. It is a microbiological preparation consisting of yeast, lactic acid bacteria, photosynthetic bacteria, *Azotobacter*, *Pseudomonas* and *Actinomycetes* and nutrients such as: potassium (3500 mg·dm^{-3}), nitrogen (1200 mg·dm^{-3}), sulphur (1000 mg·dm^{-3}), phosphorus (500 mg·dm^{-3}), sodium (200 mg·dm^{-3}), magnesium (100 mg·dm^{-3}), zinc (20 mg·dm^{-3}), manganese (0,3 mg· dm^{-3}).

Another preparation used in the experiment was a root system development stimulator, accelerating its regeneration, improving the uptake of minerals from the soil, containing 13.0% of $P_2\ 0_5$ and 5.0% of $K_2\ O$.

In the last experimental facility, an anti-stress agent containing an active and bio-available form of silicon was used to increase plant tolerance to unfavourable cultivation conditions and reduce biotic stresses caused by pathogens and pests.

The research hypothesis was adopted in the paper that natural preparations of organic and inorganic origin may have a beneficial effect on the biometric features of winter oilseed rape

plants. The aim of the research was to determine the effect of preparations containing microorganisms as well as micro and macro-elements (variant 2), phosphorus and potassium (3) and silicon (4) on the morphometric features of plants, such as: plant height before harvesting and until the first productive branching, number of productive branches and siliques on the plant, the length of the pods, the thickness of the stem at the base. The paper presents a varied reaction of winter oilseed rape morphotypes to the applied preparations and their effect in individual years of research.

MATERIAL AND METHODS

Experiment location

The research was based on a field experiment carried out in 2018-2021 at the Agricultural Experimental Station belonging to the Siedlce University of Natural Sciences and Humanities, in the climatic and soil conditions of north-eastern Poland. The experiment was set up in a split-plot system in three repetitions.

Experiment factors:

I – morphotypes of winter rape:

- population morphotype (Chrobry variety),
- hybrid morphotype with a traditional type of growth (PT 271)
- hybrid morphotype with a semi-dwarf type of growth (PX 113)

II – methods of using the preparations:

Table 1. Preparations used in the experiment

METHODS OF USING PREPARATIONS	APPLICATION DATES	DOSE
variant 1 – control object	without using the preparations	-
variant 2- organic preparation (Ugmax)	I - in autumn before sowing seeds<br>II - in spring after the start of vegetation: the beginning of the development of side shoots (BBCH 21-36)	0.9 $dm^3 \cdot ha^{-1}$
variant 3 - biostimulant containing 13.0% of $P_2 0_5$ and 5.0% of $K_2 O$	I - in autumn, 4-6 leaves phase (BBCH 13-15)<br>II – in spring after the start of vegetation (BBCH 28-30)	1.0 $dm^3 \cdot ha^{-1}$
variant 4 - biostimulant containing silicon	I - in autumn, 4-6 leaves phase (BBCH 13-15)<br>II – dense green flower bud in spring (BBCH 51)	0.5 $dm^3 \cdot ha^{-1}$

Soil conditions

The experiment was carried out on soil classified to the Haplic Luvisol group, sandy, very good rye soil complex, valuation class IVa (WRBSR, 2014). The pH of the soil was slightly

acidic and ranged from 5.68 to 5.75 in the years of research. The soil was characterized by low abundance in assimilable forms of phosphorus and medium assimilability in potassium and magnesium.

Fertilization

The winter triticale was the forecrop for winter rape in individual years of the study. Table 2 shows the applied mineral fertilization.

Table 2. Mineral fertilization used in the experiment

Date of fertilization	Type of fertilization	Doses
**before sowing**	phosphorus-potassium fertilization	40 kg P·ha^{-1}<br>110 kg K·ha^{-1}<br>first dose 40 kg N·ha^{-1}
**during autumn growth and development**	Lubofos	dose 600 kg:<br>i.e. 21 kg N·ha^{-1}<br>26.4 kg P·ha^{-1}<br>92.1 kg K·ha^{-1}<br>34.8 kg S·ha^{-1}<br>1.2 kg B·ha^{-1}
	ammonium sulphate	55.9 kg·ha^{-1} (19 kg N·ha^{-1}),
	triple superphosphate	29.6 kg·ha^{-1} (13.6 kg P·ha^{-1})
	potassium salt	29 kg·ha^{-1} (17.9 kg K·ha^{-1})
**in spring before vegetation starts (BBCH 28-30)**	ammonium nitrate	255.5 kg·ha^{-1} (86.9 kg N·ha^{-1})
	ammonium sulphate	62.5 kg·ha^{-1} (13.1 kg N·ha^{-1} +15 kg S·ha^{-1})
**at the beginning of budding (BBCH 50)**	ammonium nitrate	176.5 kg·ha^{-1} (60 kg N·ha^{-1})

Sowing

Winter rape was sown at a row spacing of 22.5 cm, maintaining the density of 45 plants per m^{-2}. The sowing was carried out at the optimal time recommended for this region (from 10 to 15 August).

Chemical protection

Chemical protection against weeds, diseases and pests was applied in accordance with the recommendations of good agricultural practice.

Table 3. Type of chemical protection treatments

Active substance in the agent used	Dose of the agent used	Development phases	acc. to the BBCH scale
		**HERBICIDES**	
clomazone	0.25 dm^3·ha^{-1}	immediately after sowing on the soil	00 BBCH
fluazifop-P-butyl	2.0 dm^3·ha^{-1}	phase of 3-4 leaves	13-14 BBCH
		**INSECTICIDES**	

thiacloprid deltamethrin	0.6 $dm^3 \cdot ha^{-1}$	1 treatment – growth of the main shoot,<br>2 treatment – development of flower buds<br>3 treatment - flowering	30 BBCH<br>50-58 BBCH<br>60-69 BBCH
		**FUNGICIDES**	
tebuconazole	0.75 $dm^3 \cdot ha^{-1}$	phase of 4-8 leaves of rape	14-18 BBCH
fluopyram prothioconazole	1.0 $dm^3 \cdot ha^{-1}$	beginning of flowering	61 BBCH
prochloraz	1.0 $dm^3 \cdot ha^{-1}$	fall phase of the first flower petals	65 BBCH

Harvest

Rapeseed was harvested at the stage of full maturity (BBCH 79), in two stages in the first and second decade of July.

Evaluation of yielding components

Immediately before harvesting (BBCH 86-87), the following elements of the yield structure were determined on a sample of 20 plants from each plot:

- plant height before harvesting (cm),
- the height of the first fruit-bearing lateral branching on the main shoot (cm),
- number of productive branches (pcs.),
- number of pods per plant (pcs.),
- number of seeds in a pod (pcs.),
- thickness of stems at the base (mm),

The number of seeds in a silique was determined on a sample of 20 siliques taken from the central part of the main shoot and side branches of a single plant.

Statistical analysis

The results of the research were statistically processed using the analysis of variance. The significance of the sources of variability was tested with the Fischer-Snedecor “F” test, and the assessment of the significance of differences at the significance level of α = 0.05 between the compared means was tested with multiple Tukey intervals.

Climatic conditions

In the years of the research differences in terms of climate were demonstrated (Table 4, 5). In the second year of the study, the highest rainfall was recorded, which was 419.0 mm on average, and the highest average air temperature was 10.1°C. It was a very wet growing season (K=2.68), with quite dry, dry, very dry months, and the period from May to July was extremely wet. In the 2018-2019 growing season, the lowest rainfall was recorded (average 244.0 mm), and the average air temperature in this growing season was 9.7°C. The last year

of the study was characterized by the lowest average air temperature of 9.3°C on average. In terms of the calculated Sielianinov coefficient, it was a very wet year of research (K=2.99).

Table 4. The value of the Sielianinow hydrothermal coefficient

	Value of the Sielianinow hydrothermal coefficient *								
	**VIII**	**IX**	**X**	**III**	**IV**	**V**	**VI**	**VII**	**Mean**
**I year of research**	1.19	1.72	2.42	3.13	0.60	4.49	1.68	1.60	2.10
**II year of research**	2.20	1.22	0.89	1.31	0.70	5.42	6.14	3.56	2.68
**III year of research**	0.90	2.50	4.39	3.55	6.36	2.38	1.66	2.20	2.99

* Coefficient value (Skowera, 2014): Extremely dry (ss) k≤0.4; Very dry (bs) 0.4-0.7; Dry (s) 0.7-1.0; Rather dry (ds) 1.0<k≤1.3; Optimal (o) 1.3<k≤1.6; Rather wet (dw) 1.6<k≤2.0; Wet (w) 2.0<k≤2.5; Very wet (bw) 2.5<k≤3.0; Extremely wet (sw) k>3.0

Table 5. Climatic data 2019-2021 (gricultural Experimental Station, Poland)

	Precipitation (mm)				Air temperature (°C)			
	**I year**	**II year**	**III year**	**Multiyear total (1996-2010)**	**I year**	**II year**	**III year**	**Multiyear mean (1996-2010)**
**VIII**	24.5	43.9	18.2	59.9	20.6	19.9	20.2	18.5
**IX**	27.4	17.4	38.8	42.3	15.9	14.2	15.5	13.5
**X**	23.3	9.5	52.7	24.2	9.6	10.7	12.0	7.9
**XI**	9.8	17.8	34.0	20.2	3.3	6.1	5.0	4.0
**XII**	9.0	29.1	34.0	18.6	0.4	2.9	-1.0	-0.1
**I**	7.9	12.9	22.6	19.0	-3.0	1.9	-1.9	-3.2
**II**	4.7	26.8	10.4	16.0	2.2	2.9	-2.5	-2.3
**III**	15.0	5.9	9.6	18.3	4.8	4.5	2.7	2.4
**IV**	5.9	6.0	42.0	33.6	9.8	8.6	6.6	8.0
**V**	59.8	63.5	29.5	58.3	13.3	11.7	12.4	13.5
**VI**	35.9	118.5	33.8	59.6	21.4	19.3	20.4	17.0
**VII**	29.7	67.7	50.0	57.5	18.5	19.0	22.7	19.7
**Mean**	**244.0**	**419.0**	**375.6**	**427.5**	**9.7**	**10.1**	**9.3**	**8.2**

RESULTS AND DISCUSSION

Based on the research, it was shown that the population morphotype was distinguished by the **highest plant height**. This value was higher by 13.7 cm on average compared to the long-stemmed hybrid (Table 6,7). However, other authors (Sikorska et al. 2018, 2019) obtained the same height in a restored hybrid with a traditional growth type and population variety. The types of preparation used significantly increased the value of this feature compared to the control variant (Table 6,7). Plants of all tested cultivars were the highest after application of the organic preparation containing microorganisms as well as micro and macro-

elements, significantly lower after the application of the preparation with silicon, and the lowest after supplementation with the preparation with 13.0% of $P_2 0_5$ and 5.0% of K_2 O. It should be emphasized that among the tested cultivars, the largest significant increase of 8.4 cm on average was shown in the population morphotype after the application of the Ugmax preparation. In the half-dwarf hybrid, the same value of this feature was found in the objects where the preparation with phosphorus and potassium and silicon were applied. Similar conclusions were reached by Matysiak and Miziniak (2019), who showed that winter rape plants after application of the mixture of Kelpak and Lithovit in the BBCH 32 phase, and twice Lithovit (BBCH 32 and 67) were characterized by an average height of 10–11% higher compared to control plants. Similar results were obtained by Przybysz et al. (2008), Sikorska et al. (2018) after using the Asahi SL biostimulant. Sikorska et al. (2019) after applying amino acids, sulphur and boron, noted a significant increase in plant height before harvest from 2.3 to 5.5 cm, but the differences between the variants were statistically insignificant. Nowak and Wenda-Piesik (2017) also found a beneficial effect of a preparation containing 19 amino acids on plant growth. Dautartė et al. (2017) after using a liquid bioorganic Raskila preparation containing a complex of macro- and trace elements, humic substances, fulvic acids, nitrogen, potassium, phosphorus, phyto-vitamins and beneficial soil microorganisms, they obtained a significant increase in plant height, on average, from 118.16 to 127.64 cm. Own research showed that the **height of the first productive branching** was the highest in the population morphotype. The highest value of this feature was also shown on object 2, where an organic preparation was used (Table 6,7). This value was higher on average by 3.3 cm compared to the control variant. Differences between the object where phosphorus and potassium and silicon were used were statistically insignificant in all tested morphotypes. The semi-dwarf hybrid had lower productive branching compared to the long-stem cultivar, while after applying the organic preparation, both hybrids showed the same reaction to the applied limited preparation. The research showed that the hybrid with the traditional type of growth had the **largest number of productive branches,** while the population form and the semi-dwarf hybrid were characterized by the same value of this feature. Significantly the highest value of this feature was recorded after the application of the organic preparation, and the lowest after the application of preparations containing phosphorus and potassium. It should be noted that the tested morphotypes had the same number of productive branches after application of Ugmax (Table 6,7). Similarly, Passandideh et al. 2021 after application of amino acids and Dautartė et al. (2017) after application of organic preparations obtained a significant increase in the value of this feature. **The number of siliques per plant** was the

highest in the long-stemmed hybrid variety, while the population form and the semi-dwarf hybrid had the same value of this feature. After the application of the organic preparation, the number of siliques on the plant increased by an average of 14.0 pcs compared to the control variant, and after the application of the preparation containing phosphorus and potassium, this value was the same as on the object where no preparations were used. It should be emphasized that in the hybrid morphotype with a traditional type of growth, after the application of the organic preparation, this value increased on average by 14.4 units, while the population form and the half-dwarf hybrid were characterized by the same value of this feature on this object (Table 6,7). Jarecki et al. 2019, after foliar application of a preparation containing Mg, B, Cu, Fe, Mn, Mo, Zn showed that it significantly increased the number of siliques on the plant compared to the control. Chwil (2016) came to similar conclusions.**The length of the pods** was the greatest in the long-stemmed hybrid, while the population variety and the semi-dwarf hybrid had the same value of this feature. On the object with the organic preparation, the plants were characterized by the greatest length of siliques, but the population form and the semi-dwarf hybrid were characterized by the same value of this feature on this object (Table 6,7). **The thickness of the stem at the base** was the greatest in the population morphotype, significantly lower in the semi-catalyst hybrid, and the smallest in the long-stemmed form (Table 6,7). Sikorska et al. (2018) came to different conclusions, who noted the greatest thickness of the stem at the base in the restored hybrids, and significantly less in the population variety. The value of this feature was the highest after using the preparation with microorganisms. On objects 3 and 4, where phosphorus, potassium and silicon were used, respectively, the same value of this feature was obtained. Climatic conditions had a significant effect on the biometric features of plants determined before harvesting. The highest values of these features in all tested cultivars were shown in the most humid year of the research. Statistically proven interaction of years and biostimulants used proves a different effect of the biostimulant in changing climatic conditions during the research (Table 6,7, 8).

Table 6. Biometric characteristics of plants depending on the factors of experience

**Methods of using preparations**	**Cultivars**			**Mean**
	population	restored hybrid with a traditional type of growth	restored hybrid with a semi-dwarf type of growth	
**Highest plant height (cm)**				
1. Variant control	133.7	120.7	119.4	**124.6**
2. Organic preparation containing	142.1	127.7	123.4	**131.1**

microorganisms as well as micro and macro elements				
3. Biostimulant containing 13.0% of P_2O_5 and 5.0% of potassium oxide (K_2O)	137.0	123.4	121.0	**127.4**
4. Biostimulant containing silicon	139.2	131.1	122.0	**128.6**
**Mean**	**138.0**	**124.3**	**121.5**	-
			**$LSD_{0.05}$ for:**	
			cultivars	0.6
			methods of using preparations	0.8
			interaction: cultivars x methods of using preparations	1.4
**Height of the first productive branching (cm)**				
1. Variant control	45.4	38.0	36.8	**40.1**
2. Organic preparation containing microorganisms as well as micro and macro elements	49.1	40.9	40.2	**43.4**
3. Biostimulant containing 13.0% of P_2O_5 and 5.0% of potassium oxide (K_2O)	47.9	40.1	38.9	**42.3**
4. Biostimulant containing silicon	49.0	40.3	39.1	**42.8**
**Mean**	**47.8**	**39.8**	**38.8**	-
			**$LSD_{0.05}$ for:**	
			cultivars	0.5
			methods of using preparations	0.7
			interaction: cultivars x methods of using preparations	1.2
**Largest number of productive branches (pcs.)**				
1. Variant control	3.9	4.2	4.0	**4.0**
2. Organic preparation containing microorganisms as well as micro and macro elements	5.1	5.1	5.0	**5.1**
3. Biostimulant containing 13.0% of P_2O_5 and 5.0% of potassium oxide (K_2O)	4.1	4.5	4.1	**4.2**
4. Biostimulant containing silicon	4.5	4.7	4.7	**4.6**
**Mean**	**4.4**	**4.6**	**4.4**	**-**
			**$LSD_{0.05}$ for:**	
			cultivars	0.1
			methods of using preparations	0.1
			interaction: cultivars x methods of using preparations	0.2
**The number of siliques per plant (pcs.)**				
1. Variant control	130.6	136.8	128.9	**132.1**
2. Organic preparation containing microorganisms as well as micro and macro elements	143.6	150.2	144.2	**146.0**
3. Biostimulant containing 13.0% of P_2O_5 and 5.0% of potassium oxide (K_2O)	131.2	133.1	132.0	**132.1**
4. Biostimulant containing silicon	139.3	145.8	139.6	**141.6**

	**Mean**	**136.2**	**141.5**	**136.2**	**-**
				**LSD0.05 for:**	
				cultivars	1.7
				methods of using preparations	1.5
				interaction: cultivars x methods of using preparations	2.6
**The length of the pods (cm)**					
1.	Variant control	7.0	7.2	6.9	**7.0**
2.	Organic preparation containing microorganisms as well as micro and macro elements	7.8	8.1	7.8	**7.9**
3.	Biostimulant containing 13.0% of $P_2 0_5$ and 5.0% of potassium oxide (K_2O)	7.0	7.5	7.0	**7.2**
4.	Biostimulant containing silicon	7.5	7.7	7.5	**7.6**
	**Mean**	**7.3**	**7.6**	**7.3**	**-**
				**LSD0.05 for:**	
				Cultivars	0.1
				methods of using preparations	0.1
				interaction: odmiany x methods of using preparations	0.1
**The thickness of the stem at the base (mm)**					
1.	Variant control	13.80	13.62	13.91	**13.78**
2.	Organic preparation containing microorganisms as well as micro and macro elements	15.04	14.39	15.21	**14.88**
3.	Biostimulant containing 13.0% of $P_2 0_5$ and 5.0% of potassium oxide (K_2O)	14.82	13.98	14.11	**14.30**
4.	Biostimulant containing silicon	14.68	13.97	14.24	**14.30**
	**Mean**	**14.59**	**13.99**	**14.37**	**-**
				**LSD0.05 for:**	
				cultivars	0.17
				methods of using preparations	0.24
				interaction: cultivars x methods of using preparations	0.42

Table 7. Biometric characteristics of plants depending on years of research and morphotypes

**Years**	**Cultivars**			**Mean**
	population	restored hybrid with a traditional type of growth	restored hybrid with a semi-dwarf type of growth	
**Highest plant height (cm)**				
I	131.5	121.5	120.0	**124.3**
II	142.8	126.8	122.0	**130.5**
III	139.8	124.6	122.3	**128.9**
**Mean**	**138.0**	**124.3**	**121.5**	**-**
			**LSD0.05 for:**	
			cultivars	0.6
			years	0.6

			interaction: cultivars x years	1.1
**Height of the first productive branching (cm)**				
I	40.7	35.7	34.1	**36.8**
II	57.2	43.4	42.5	**47.7**
III	45.6	40.3	39.7	**41.9**
**Mean**	**47.8**	**39.8**	**38.8**	-
			**$LSD_{0.05}$ for:**	
			cultivars	0.5
			years	0.5
			interaction: cultivars x years	0.8
**Largest number of productive branches (pcs.)**				
I	3.9	3.9	3.7	**3.8**
II	5.0	5.4	5.2	**5.2**
III	4.4	4.6	4.5	**4.5**
**Mean**	**4.4**	**4.6**	**4.4**	**-**
			**$LSD_{0.05}$ for:**	
			cultivars	0.1
			years	0.1
			interaction: cultivars x years	0.2
**The number of siliques per plant (pcs.)**				
I	125.9	130.9	124.8	**127.2**
II	150.4	148.8	146.9	**148.7**
III	132.2	144.6	136.9	**137.9**
**Mean**	**136.2**	**141.5**	**136.2**	**-**
			**$LSD_{0.05}$ for:**	
			cultivars	1.7
			years	1.7
			interaction: cultivars x years	2.9
**The length of the pods (cm)**				
I	6.7	6.9	6.6	**6.7**
II	8.2	8.5	7.9	**8.2**
III	7.1	7.6	7.4	**7.3**
**Mean**	**7.3**	**7.6**	**7.3**	**-**
			**$LSD_{0.05}$ for:**	
			cultivars	0.1
			years	0.1
			interaction: cultivars x years	0.2
**The thickness of the stem at the base (mm)**				
I	13.63	13.04	13.66	**13.44**
II	15.74	15.18	15.48	**15.47**
III	14.39	13.75	13.97	**14.04**
**Mean**	**14.59**	**13.99**	**14.37**	**-**
			**$LSD_{0.05}$ for:**	
			cultivars	0.17
			years	0.17
			interaction: cultivars x years	0.29

Table 8. Biometric characteristics of plants depending on the years of research and the types of preparations used

Methods of using preparations	*Years*			Mean
	**2018-2019**	**2019-2020**	**2020-2021**	
**Highest plant height (cm)**				
1. Variant control	120.4	127.3	126.2	**124.6**
2. Organic preparation containing microorganisms as well as micro and macro elements	128.0	133.6	131.6	**131.1**
3. Biostimulant containing 13.0% of $P_2 0_5$ and 5.0% of potassium oxide (K_2O)	123.6	129.7	128.9	**127.4**
4. Biostimulant containing silicon	125.2	131.5	129.0	**128.6**
**Mean**	**124.3**	**130.5**	**128.9**	**-**
**$LSD_{0.05}$ for:**				
years				0.6
methods of using preparations				0.8
interaction: *years* x *methods of using preparations*				1.4
**Height of the first productive branching (cm)**				
1. Variant control	34.5	46.2	39.5	**40.1**
2. Organic preparation containing microorganisms as well as micro and macro elements	38.3	48.6	43.2	**43.4**
3. Biostimulant containing 13.0% of $P_2 0_5$ and 5.0% of potassium oxide (K_2O)	37.1	47.6	42.2	**42.3**
4. Biostimulant containing silicon	37.4	48.4	42.6	**42.8**
**Mean**	**36.8**	**47.7**	**41.9**	-
**$LSD_{0.05}$ for:**				
years				0.5
methods of using preparations				0.7
interaction: *years* x *methods of using preparations*				r.n.
**Largest number of productive branches (pcs.)**				
1. Variant control	3.3	4.7	4.1	**4.0**
2. Organic preparation containing microorganisms as well as micro and macro elements	4.4	5.9	5.0	**5.1**
3. Biostimulant containing 13.0% of $P_2 0_5$ and 5.0% of potassium oxide (K_2O)	3.4	4.7	4.6	**4.3**
4. Biostimulant containing silicon	4.2	5.4	4.3	**4.6**
**Mean**	**3.8**	**5.2**	**4.5**	**-**
**$LSD_{0.05}$ for:**				
years				0.1
methods of using preparations				0.1
interaction: *years* x *methods of using preparations*				0.2
**The number of siliques per plant (pcs.)**				
1. Variant control	122.2	144.0	130.1	**132.1**
2. Organic preparation containing	134.1	154.9	148.9	**146.0**

microorganisms as well as micro and macro elements				
3. Biostimulant containing 13.0% of $P_2 0_5$ and 5.0% of potassium oxide ($K_2 O$)	121.9	143.6	130.8	**132.1**
4. Biostimulant containing silicon	130.6	152.3	141.7	**141.6**
**Mean**	**127.2**	**148.7**	**137.9**	**-**
			**LSD0.05 for:**	
			years	1.7
			methods of using preparations	1.5
			interaction: *years* x *methods of using preparations*	2.6
**The length of the pods (cm)**				
1. Variant control	6.2	8.0	7.0	**7.0**
2. Organic preparation containing microorganisms as well as micro and macro elements	7.3	8.5	7.8	**7.9**
3. Biostimulant containing 13.0% of $P_2 0_5$ and 5.0% of potassium oxide ($K_2 O$)	6.3	7.9	7.2	**7.2**
4. Biostimulant containing silicon	7.1	8.4	7.4	**7.6**
**Mean**	**6.7**	**8.2**	**7.3**	**-**
			**LSD0.05 for:**	
			years	0.1
			methods of using preparations	0.1
			interaction: *years* x *methods of using preparations*	0.1
**The thickness of the stem at the base (mm)**				
1. Variant control	12.86	14.90	13.58	**13.78**
2. Organic preparation containing microorganisms as well as micro and macro elements	14.19	15.98	14.48	**14.88**
3. Biostimulant containing 13.0% of $P_2 0_5$ and 5.0% of potassium oxide ($K_2 O$);	13.38	15.42	14.11	**14.30**
4. Biostimulant containing silicon	13.34	15.57	13.98	**14.30**
**Mean**	**13.44**	**15.47**	**14.04**	**-**
			**LSD0.05 for:**	
			years	0.17
			methods of using preparations	0.24
			interaction: *years* x *methods of using preparations*	0.42

CONCLUSIONS

1. The research showed that the population morphotype was characterized by the highest plant height before harvesting, the height to the first branching, the thickness of the stem at the base, and the long-stemmed hybrid had the largest number of productive branches and siliques on the plant.

2. The organic preparation containing microorganisms as well as micro and macro-elements, applied in the autumn before sowing seeds and in the spring after the start of vegetation, had the most beneficial effect on the biometric characteristics of plants before harvesting.
3. The weather conditions accompanying the vegetation of the crop has the decisive influence on the biostimulating effect of the applied preparations, in particular the amount and distribution of rainfall. The positive effect of the preparations on the biometric features of plants was revealed in the most humid year of the research.

REFERENCES

1. Al-Taweil H.I., Bin Osman M., Hamid A.A., Yusoff W.M. 2009. Development of microbial inoculants and the impact of soil application on rice seedlings growth. Am. J. Agric. Biol. Sci., 4, 79–82.
2. Chwil S. 2016. The effect of foliar feeding under different soil fertilization conditions on the yield structure and quality of winternoilseed rape (*Brassica napus* L.). EJPAU, 19: #02
3. Du Jardin, P. 2015. Plant biostimulants: Definition. concept, main categories and regulation. Sci. Hort., 196, 3-14.
4. Jarecki W., Buczek J., Bobrecka-Jamro D. 2019. The response of winter oilseed rape to diverse foliar fertilization. Plant Soil Environ., 65, 125-130.
5. Dautartė A., Spruogis V., Zemeckis R., Bartkevičius E., Gavenauskas A. 2017. The influence of bioorganic preparations on the productivity of conventionaly grown winter rape activating and saving the use of synthetic chemicals. Proceedings of the 8th International Scientific Conference Rural Development 2017 Edited by prof. Asta Raupelienė. DOI: org/10.15544/RD.2017.051
6. Findura P, Šindelková I, Rusinek R, Karami H, Gancarz M, Bartoš P. 2022. Determination of the influence of biostimulants on soil properties and field crop yields. Int. Agrophys., 36(4), 351-359. DOI:10.31545/intagr/155955.
7. Gobin A. 2012. Impact of heat and drought stress on arable crop production in Belgium. Natural Hazards and Earth System Sci., 12(6), 1911-1922. DOI: org/10.5194/nhess-12-1911-2012
8. Matysiak K., Miziniak W. 2019. Assessment of the biostimulative effect of Lithovit and Kelpak on selected biometric and qualitative features of winter oilseed rape. Progress in Plant Protection, 59, (3), 188-195. DOI: 10.14199/ppp-2019-025

(in Polish).

9. Mrid R.B., Benmrid B., Hafsa J., Boukcim H., Sobeh M., Yasri A. 2021. Secondary metabolites as biostimulant and bioprotectant agents: A review, Science of The Total Environment, 777, 146-204, ISSN 0048-9697, DOI: org/10.1016/j.scitotenv.2021.146204.
10. Nowak A., Wenda-Piesik A. 2014. Effects of microelements and amino acids apllication on winter rape plant and canopy. 13th ESA Congress 25-29 August, Debrecen, Hungary
11. Passandideh M., Rajaei M., Zeinalzadeh-Tabrizi H. 2021. Effect of some plant growth biostimulants on increasing canola (*Brassica napus* L.) tolerance to drought stress. Environmental Stresses in Crop Sciences 15, No. 4, pp. 1023-1035 (Winter 2023) doi.org/10.22077/escs.2021.4209.1988
12. Pietkiewicz S., Kołpak R., Wietrzyńska A., Łoboda T., Ostrowska D., 2004. Wpływ efektywnych mikroorganizmów na wzrost i plonowanie ziemniaków rozmnażanych z minibulw. Rocz. Gleb. 60(1), 285–290.
13. Przybysz, A., Małecka-Przybysz, M., Słowiński, A., Gawrońska, H. 2008. The effect of Asahi SL on growth, efficiency of photosynthetic apparatus and yield of field grown oilseed rape. in Monographs Series: Biostimulators in Modern Agriculture: Field Crops, ed Dąbrowski Z. T., editor. (Warsaw: Editorial House Wieś Jutra;), 7-17.
14. Rutkowska, A.; Pikuła, D.2013. Effect of crop rotation and nitrogen fertilization on the quality and quantity of soil organic matter. Soil Process. Curr. Trends Qual. Assess.,249–267.
15. Sikorska A., Gugała M., Zarzecka K. 2019. The impact of different types of foliar feeding on the architecture elements of a winter rape (*Brassica napus* L.) field, Applied Ecology and Environmental Research, 18(1), 263-273. DOI: 10.15666/aeer/1801_263273.
16. Sikorska, A., Gugała, M., Zarzecka, K. 2018. Response of winter rapeseed to biostimulator application and sowing method. Part I. Field architecture elements. Acta Sci. Pol. Agricultura, 17(4), 205-214.
17. Skowera B. 2014. Changes of hydrothermal conditions in the Polish area (1971–2010). Fragm. Agron., 31, 74–87. (In Polish)
18. World Reference Base for Soil Resources. International Soil Classification System for Naming Soils and Creating Legends for Soil. In World Soil Resources Reports; Field

Experiment; Food and Agriculture Organization: Rome, Italy, 2014. Available online: http://www.fao.org (accessed on 1 February 2023).

19. Vessey J.K. 2003. Plant growth promoting rhizobacteria as biofertilizer. Plant Soil 255, 571-586.
