# Supplementary material for: Biometric characteristics of winter rape plants (Brassica napus L.) before harvest in the soil and climatic conditions of north-eastern Poland
Source: PLoS One. 2023 Aug 16;18(8):e0289947. doi: 10.1371/journal.pone.0289947 (PMC10431616; doi:10.1371/journal.pone.0289947)
Supplement: Attachment — Submitted filename: comments PONE-D-23-15441.docx [file pone.0289947.s010.docx]

**Comments to Author**

**Article Title:** Biometric characteristics of winter rape plants (Brassica napus l.) before harvest in the soil and climatic conditions of north-eastern Poland, depending on the bio-stimulative preparations used

**Summary:** I have reviewed the manuscript and it is very interesting. Dear Editor, this publication will provides new insight about the bio-stimulants and the organic preparation containing microorganisms as well as micro and macro-elements, applied in the autumn before sowing of seeds and in the spring after the start of vegetation, had the most beneficial effect on the biometric characteristics of rape plants before harvesting. I therefore, suggest the **minor revision** for this manuscript and **recommend it for publication**.

**Comments:**

**Abstract**

It’s better to include the data in form of percentage for different parameters/observations under this study in autum and spring.

**Introduction**

Introduction is well written and have all the necessary ingredients. Please, read the manuscript carefully and make necessary amendments e.g. spell mistakes

**Material and Methods**

Add data regarding nutrient analysis of soil before and after the crop grown to kmow the impact of biostimulants.

**Results and Discussion**

Results and discussion part is well written with logical explanation. However, add few more recent references in the discussion part.

**Conclusion**

Conclusion is decent.

**References**

Please check the references carefully and follow journal guidelines.
