# Supplementary material for: Biometric characteristics of winter rape plants (Brassica napus L.) before harvest in the soil and climatic conditions of north-eastern Poland
Source: PLoS One. 2023 Aug 16;18(8):e0289947. doi: 10.1371/journal.pone.0289947 (PMC10431616; doi:10.1371/journal.pone.0289947)
Supplement: Attachment — Submitted filename: Response to Reviewers.docx [file pone.0289947.s012.docx]

Response to Reviewers

Dear Reviewers, thank you very much for your valuable substantive comments. Corrections were made in the article (the changes were made in red font) in accordance with the reviewers’ suggestions, e.g.

- The title was changed from ”*Biometric characteristics of winter rape plants (Brassica napus l.) before harvest in the soil and climatic conditions of north-eastern Poland, depending on the bio-stimulative preparations used*” to “*Biometric characteristics of winter rape plants (Brassica napus l.) before harvest in the soil and climatic conditions of north-eastern Poland*”
- errors in citations have been corrected in the text and missing references to literature have been supplemented in the text. In the section on materials and methods, missing references to tables were added, years of research were specified, software used, chemical protection applied was clarified. Research results and discussion were corrected.
- The article was corrected in accordance with the editorial requirements of the journal.
- As suggested by the reviewer, the tested parameters were changed in the appropriate order, i.e. the height of the first fruit-bearing lateral branching on the main shoot, the thickness of the stem at the base, number of productive branches and siliques on the plant, the length of the pods, plant height before harvesting.
- I would like to inform you, as I also included in the article, that conducting the field experiment did not require obtaining any permits for field work, because the Zawady Agricultural Experimental Station is part of the University of Natural Sciences and Humanities in Siedlce. Field experiments have been conducted in the Zawady experimental station for over 40 years.
- Major and minor experimental factors are listed as suggested,
- According to the guidelines, the characteristics of the preparations used were placed in the Materials and Methods section,
- The section on chemical protection describes good agricultural practices in chemical protection of plants.
- The text indicates that the statistical calculations were made on the basis of a proprietary algorithm written in Excel in accordance with a mathematical model.
